# Changes in Thyroglobulin Antibody Levels in Differentiated Thyroid Cancer Patients After Thyroidectomy: A Retrospective Study in Basrah, Iraq

**DOI:** 10.7759/cureus.66557

**Published:** 2024-08-10

**Authors:** Mustafa M Jebur, Ibrahim H Hussein, Haider A Alidrisi, Abbas A Mansour

**Affiliations:** 1 Diabetes and Endocrinology, Faiha Specialized Diabetes, Endocrine, and Metabolism Center, Basrah, IRQ; 2 Diabetes and Endocrinology, College of Medicine, University of Basrah, Basrah, IRQ; 3 Endocrinology, Faiha Specialized Diabetes, Endocrine, and Metabolism Center, Basrah, IRQ; 4 Endocrinology, College of Medicine, University of Basrah, Basrah, IRQ

**Keywords:** thyroid cancer, changes of antithyroglobulin antibody, thyroid cancer recurrence, thyroglobulin antibodies (tgab), differentiated thyroid cancer (dtc)

## Abstract

Introduction: Differentiated thyroid cancer (DTC), the most common endocrine malignancy is subdivided into papillary (the most common) and follicular type. Generally, DTC has a good prognosis with standard treatments such as surgery and, in some cases, radioactive iodine (RAI). Post-treatment follow-up includes thyroglobulin (Tg) and anti-thyroglobulin antibody (TgAb) measurement and imaging to assess treatment success and detect recurrence. However, TgAb can interfere with Tg measurements, making it essential to measure TgAb at different times (months).

Aim of the study: The aim of this study was to evaluate the changes in TgAb level in DTC patients after thyroidectomy and its association with recurrence.

Methods: This was a retrospective cohort study done at the Faiha Specialized Diabetes, Endocrine, and Metabolism Center (FDEMC), Basrah, Iraq, for individuals diagnosed with DTC between 2008 and 2023. The data collected were analyzed using IBM SPSS Statistics for Windows, Version 21.0 (Released 2012; IBM Corp., Armonk, New York, United States). The categories were classified according to the TgAb level as: (i) elevated (>115 IU/ml) and (ii) normal (<115 IU/ml), where TgAb levels measured at 0-6 months, 6-12 months, 24-36 months, 36-48 months, and beyond 48 months.

Results: The mean age at diagnosis of the study population (n=108) was 40.15 years with a female-to-male ratio of 4:1. Among these individuals, 52.8% (n=57) were found to be obese. Total thyroidectomy was performed on 84.3% (n=91). Papillary thyroid cancer was diagnosed in 69.5% (n=75). TgAb levels were influenced by body mass index (BMI); higher BMI (>30kg/m^2^) was associated with less consistent TgAb normalization, particularly beyond 48 months (P = 0.04). The study found no significant differences in TgAb normalization based on gender, age, BMI, type of surgery, type of cancer, American Thyroid Association (ATA) risk of recurrence, or radioactive iodine (RAI) treatment.

Conclusion: Factors including gender, age, type of surgery, type of cancer, ATA risk of recurrence, and RAI treatment did not significantly affect TgAb normalization in DTC individuals over the study period. However, higher BMI is associated with less consistent TgAb normalization in the long term.

## Introduction

Differentiated thyroid cancer (DTC) is the most prevalent endocrine malignancy, accounting for over 85% of thyroid cancer cases. The majority of these cases are papillary thyroid cancer, with a smaller proportion being follicular thyroid cancer [1]. Fortunately, they have a good prognosis if managed appropriately. Standard management of DTC includes surgery with either total thyroidectomy or lobectomy in low-risk cases in addition to RAI when required [2].

Thyrotropin or thyroid-stimulating hormone (TSH) is secreted by the pituitary gland and stimulates thyroid gland growth through its receptors, playing a significant role in thyroid cancer risk [3]. The rate of thyroid cancer recurrence is more than three times higher when TSH levels exceed 4 mIU/L compared to when TSH levels are low [4]. Long-term treatment with levothyroxine is used to suppress TSH levels, thereby preventing its stimulatory effects on thyroid cells and inhibiting regrowth [5].

Although both normal and malignant thyroid cells produce serum thyroglobulin (Tg), Tg is not suitable for diagnosing DTC. However, following thyroidectomy or RAI therapy, the lowest serum Tg can serve as a long-term tumor marker for monitoring patients [6]. After surgery, monitoring levels of Tg are typically observed within three to four weeks, although it may take several months for Tg to disappear from the serum following RAI treatment or total thyroidectomy [7]. Monitoring serum Tg levels postoperatively is crucial for assessing evidence of biochemical recurrence or not and therefore success of treatment [8].

Tg levels can be measured while TSH is suppressed by levothyroxine therapy withdrawal. Similarly, levels can also be measured following TSH stimulation to elevate serum TSH above 30 mIU/L [6]. Tg concentration can be increased significantly by TSH stimulation in both normal and thyroid cancer tissue. Therefore, measuring serum TSH alongside serum Tg is essential for accurate interpretation [5].

Antithyroglobulin antibodies (TgAb) are autoantibodies targeting Tg, a glycoprotein produced by thyroid cells and subsequently inserted into the follicular lumen of the thyroid gland [8]. TgAb can interfere with the measurement of serum Tg, potentially leading to false-negative results. The presence of TgAb is significant in the context of DTC follow-up because it complicates the interpretation of serum Tg levels. When TgAb are present, they can bind to Tg, making Tg levels appear lower than they actually are, which could mask the detection of residual or recurrent disease. TgAb can also cause interference in immunoassays by either hindering Tg detection or, in some cases, falsely elevating measured Tg levels through assay interference mechanisms. Therefore, TgAb levels should be measured concurrently with Tg to ensure accurate monitoring [9].

By considering both Tg and TgAb levels, clinicians can more accurately assess the presence of thyroid tissue and the effectiveness of treatments. This dual measurement approach is crucial for the long-term monitoring and management of patients with DTC, ensuring timely detection of recurrence and appropriate intervention [9]. The aim of this study was to evaluate the changes in TgAb level in DTC patients after thyroidectomy and its association with recurrence.

## Materials and methods

Study design

**Figure 1:**
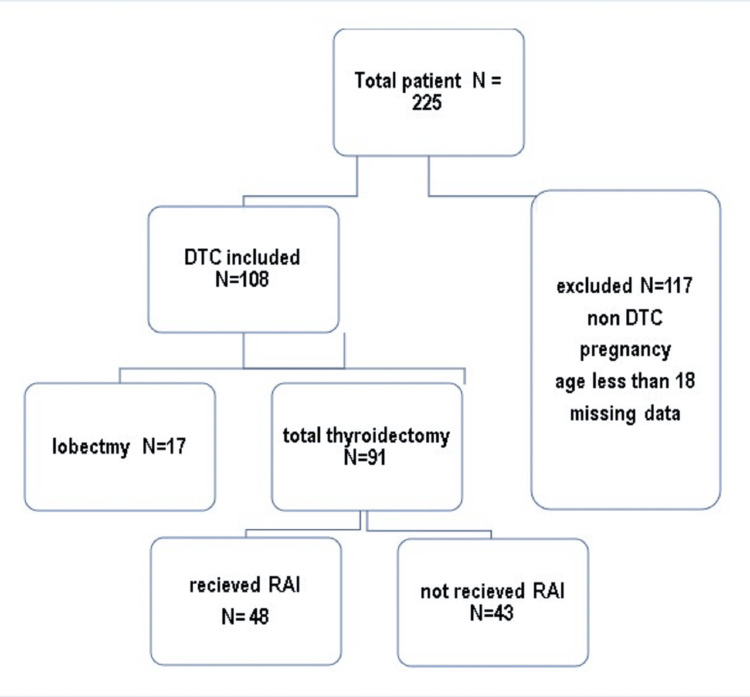
Flowchart showing inclusion and exclusion criteria with surgery type and radioactive iodine status. DTC: differentiated thyroid cancer; RAI: radioactive iodine

This was a retrospective cohort study conducted at the Faiha Specialized Diabetes, Endocrine and Metabolism Center (FDEMC), Basrah, Iraq, for patients diagnosed with DTC between 2008 and 2023. The patients included had confirmed DTC by postoperative histopathology and were over 18 years of age. We exclude patients aged less than 18 years, with non-DTC, pregnancy, and missing data as shown in Figure 1.

Clinical procedures

Patients were selected depending on whether they had DTC diagnosed by histopathology postoperatively, the type of surgery, and whether they received RAI or not. Categories were divided into two groups based on TgAb levels: elevated (>115 IU/ml) and normal (≤115 IU/ml). The disease was staged according to the American Joint Committee on Cancer (AJCC) [10]. Treatments followed the American Thyroid Association (ATA) guidelines 2015, involving lobectomy or total thyroidectomy, with lymph node dissection, when necessary, levothyroxine suppression therapy, and RAI (I131) post surgery [2].

Data collection

The data collected in this study was age, age at diagnosis, gender, body mass index, and family history of DTC. Laboratory results during follow-up comprised serum TSH, free thyroxin, Tg, and TgAb levels measured at 0-6 months, 6-24 months, 24-36 months, 36-48 months, and beyond 48 months. Recurrence was assessed using ultrasound imaging performed with Affiniti 50 G (Koninklijke Philips N.V., Amsterdam, Netherlands) with probe L12-4 done by an endocrinologist specializing in thyroid ultrasound, whole-body scans, and tissue samples from fine-needle aspiration biopsies or reoperations.

Laboratory methods

Tg measurements were performed using the chemiluminescent immunoassay Cobas® e411 platform (F. Hoffmann-La Roche AG, Basel, Switzerland). Reference values according to the kit device are as follows: TSH, 0.27-4.2 µIU/mL; Free T4, 0.93-1.7 ng/dL; Tg, 1.4-78 IU/mL; TgAb, 0-115 IU/mL. Participants were divided into two groups based on TgAb levels: (i) elevated (>115 IU/ml) and (ii) normal (≤115 IU/ml), according to the kit device.

Statistical analysis

Data were analyzed using IBM SPSS Statistics for Windows, Version 21.0 (Released 2012; IBM Corp., Armonk, New York, United States). Continuous variables such as age, BMI, and TgAb were summarized using mean, standard deviation (SD), median, and interquartile range (IQR). Categorical variables like gender, clinical presentation, and treatment type were summarized using frequency and percentage. The Chi-square test was used to assess the association between TgAb status and recurrence rates.

Ethical considerations

The study was approved by the FDEMC Research Committee (approval number: #56/35/32). Direct informed consent from each patient was not required due to the retrospective nature of the study. Confidentiality and data anonymity were rigorously maintained throughout the research according to the Helsinki Agreement and its later amendments or comparable ethical standards.

## Results

A total of 108 patients diagnosed with DTC were included in the study, with a mean age at diagnosis of 40.15 years (SD = 11.20) and an average BMI of 30.68kg/m^2^ (SD = 5.89) (Table 1). Women constituted about 81.5% of the cohort (n=88). Regarding surgical interventions, 84.3% (n=91) underwent a total thyroidectomy, and 15.7% (n=17) had a lobectomy. The most common type of cancer was papillary, affecting 69.5% (n=75), with non-papillary types in 30.5% (n=33).

**Table 1:** Demographic and clinical characteristics of the study cohort (N=108) DTC: differentiated thyroid cancer; AJCC: American Joint Committee on Cancer; ATA: American Thyroid Association; RAI: Radioactive Iodine

Variables		Mean ± SD	Number (Percentage)
Age at diagnosis (years)		40.15±11.20	
Gender	Female		88 (81.5%)
	Male		20 (18.5%)
Body mass index (kg/m^2^)		30.68±5.89	
Family history of positive DTC			7 (6.5%)
Type of surgery	Lobectomy		17 (15.7%)
	Total thyroidectomy		91 (84.3%)
Type of cancer	Papillary		75 (69.5%)
	Non-papillary		33 (30.5%)
AJCC stage	Stage 1		98 (90.7%)
	Stage 2		6 (5.5%)
ATA risk recurrence	Low		76 (70.4%)
	Intermediate		20 (18.5%)
	High		8 (7.4%)
RAI	Received		48 (44.4%)
	Not received		60 (55.6%)

The ATA risk of recurrence staging system was classified as low in 70.4% (n=76), intermediate in 18.5% (n=20), and high in 7.4% (n=8). Most patients were diagnosed at an early stage, with 90.7% (n=98) at AJCC stage 1 and 5.5% (n=6) at stage 2. Additionally, 6.5% (n=7) reported a positive family history of DTC in first- and second-degree relatives. In terms of treatment, 44.4% (n=48) received RAI therapy, while 55.6% (n=60) did not.

Table 2 presents data on factors associated with TgAb levels across different categories, including age at diagnosis, gender, BMI, type of surgery, type of cancer, ATA risk of recurrence, RAI treatment, and AJCC staging. Comparisons between men and women across different time intervals show slightly lower percentages of normal TgAb in men, although not statistically significant (p > 0.05).

**Table 2:** Analysis of changes in normal TgAb levels by various factors over time BMI: body mass index; ATA: American Thyroid Association; RAI: radioactive iodine; TgAb: anti-thyroglobulin antibody Note: There was no specific guideline for the timeframe. The comparisons were made at different follow-up times: (0-6 months, 6-12 months, 12-24 months, 24-36 months, 36-48 months, and >48 months)

Factors		Normal TgAb, n (%)					
		0-6 months (n=32)	6-12 months (n=50)	12-24 months (n=29)	24-36 months (n=23)	36-48 months (n=19)	>48 months (n=29)
Gender	Male	8 (100)	8 (72.3)	6 (100)	6 (100)	4 (80)	7 (87.5)
	Female	24 (82.8)	42 (80.8)	23 (85.2)	17 (89.5)	15 (88.2)	22 (84.6)
	p-value	0.5	0.6	1.0	1.0	1.0	1.0
BMI (kg/m^2^)	<30	13 (76.5)	24 (75)	16 (84.2)	12 (100)	11 (100)	16 (100)
	>30	19 (95)	26 (83.9)	13 (92.9)	11 (84.6)	8 (72.7)	13 (72.2)
	p-value	0.1	0.5	0.62	0.48	0.2	0.04
Age (years)	<55	29 (85.3)	42(80.8)	25 (86)	18 (90)	14 (93.3)	23 (88.5)
	>55	3 (100)	8 (72.7)	4 (100)	5 (100)	5 (71.4)	6 (75)
	p-value	1.00	0.68	1.0	1.0	0.2	0.57
Type of surgery	Lobectomy	8 (100)	13 (81.3)	3 (75)	2 (100)	2 (100)	2 (100)
	Total thyroidectomy	24 (82.8)	37 (78.7)	26 (89.7)	21 (91.3)	17 (85)	27 (84.4)
	p-value	0.5	1	0.4	0.5	1	1
Type of cancer	Papillary	29 (85.3)	46 (79.3)	26 (86.7)	20 (90.9)	15 (83.3)	25 (83.3)
	Non-papillary	3 (100)	4 (80)	3 (100)	3 (100)	4 (100)	4 (100)
	p-value	1	1	1	1	1	1
ATA risk of recurrence	Low	21 (95.5)	36 (76.6)	19 (82.6)	16 (94.1)	14 (82.4)	19 (82.6)
	Intermediate -high	11 (73.3)	12 (85.7)	9 (100)	6 (85.7)	5 (100)	9 (90)
	p-value	0.13	0.7	0.3	0.5	1	1
RAI	Not received	23 (95.8)	34 (85)	14 (82.4)	9 (100)	7 (100)	11 (100)
	Received	9 (75)	16 (69.6)	15 (93.8)	14 (87.5)	12 (80)	18 (78.3)
	p-value	0.61	0.1	0.6	0.5	0.5	0.1

Patients under 55 years of age tend to have higher percentages of normal TgAb compared to those over 55 years, without statistical significance (p > 0.05), indicating no clear age-related effect on TgAb normalization.

Comparisons between individuals with BMI <30 kg/m^2^ (n= 51, 47.2%) and those with BMI >30kg/m^2^ (n=57, 52.8%) reveal that those with BMI <30 kg/m^2^ generally have higher percentages of normal TgAb, with statistical significance at > 48 months (p = 0.04), suggesting a potential long-term association between lower BMI and better TgAb normalization which is barely significant given that it has not been statistically significant for the past four years. Therefore, to make a definitive conclusion based on BMI (obesity vs non-obesity) in relationship to TgAb normalization is not a strong argument.

Comparison between lobectomy and total thyroidectomy shows similar percentages of normal TgAb with no statistically significant differences (p > 0.05), suggesting that the type of surgery does not significantly impact TgAb normalization.

Papillary and non-papillary thyroid cancer patients exhibit comparable percentages of normal TgAb, without significant differences (all P values = 1.0), indicating that the type of cancer does not strongly affect TgAb normalization.

Lower ATA risk patients tend to have higher percentages of normal TgAb, especially noticeable in the 0-6 months period (p = 0.13) but not statistically significant, suggesting a potential association between lower risk of recurrence and early TgAb normalization. Patients not treated with RAI generally show higher percentages of normal TgAb, particularly noticeable in the 6-12 months period (p-value = 0.1) suggesting a potential association between RAI therapy and slower TgAb normalization.

**Figure 2:**
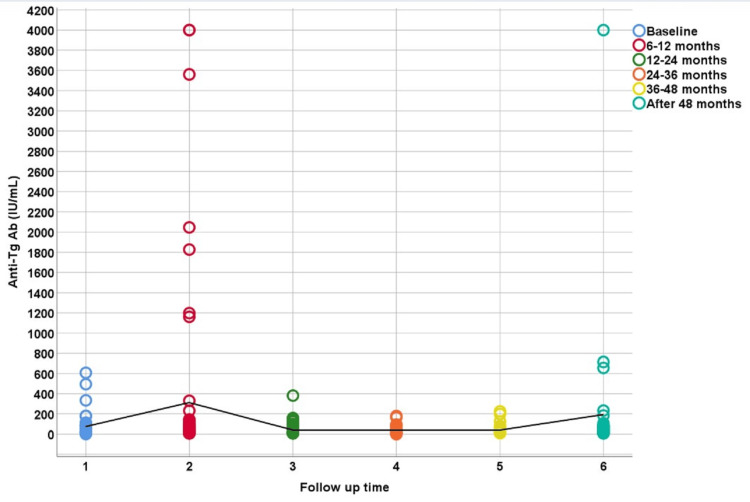
Changes in antithyroglobulin antibodies at various times (months) Baseline: 0-6 months

Figure 2 illustrates the variation in TgAb (IU/mL) levels across different time intervals for patients, depicted by colored circles. Initially, at baseline, TgAb levels are generally below 600 IU/mL. However, at the 6-12-month mark, several patients exhibit notably higher levels, reaching up to 4200 IU/mL in some cases. Subsequently, TgAb levels tend to decline, with most follow-up periods showing levels predominantly under 600 IU/mL. Although occasional spikes occur in later follow-up periods, they are less frequent and lower in magnitude compared to the 6-12 months period. Overall, the highest TgAb levels are observed at 6-12 months, followed by a general decline in subsequent periods. The black line likely indicates a median across the time points.

## Discussion

This study shows individuals with relatively young age (mean age 40.15 years) that are consistent with similar epidemiology [10], showing that thyroid cancer affects individuals in their 30s and 40s. The prevalence of DTC was higher in females in comparison to males (F:M ratio =4:1). The reproductive age factor and hormonal changes may explain this discrepancy and its similarity in nearby regions like Iran (F:M ratio =3:1) [11,12].

The patients in this study were obese with high BMI, which has been recognized as a risk factor for different types of cancer including DTC. This association may be affected by hormonal imbalances and chronic inflammatory processes [13].

There was a small percentage with a positive family history of DTC first- and second-degree relatives while most cases are sporadic [14]. This means that there is a need for genetic screening for patients with positive family history [15].

Regarding the surgical intervention, the majority underwent total thyroidectomy while a small percentage did lobectomy. Total thyroidectomy was preferred in those with confirmed malignancy or a high recurrent rate, removing the essential disease sites and facilitating RAI therapy [2]. The most common type of DTC in this study was papillary, which is similar to global data and has a good prognosis with high survival [16].

AJCC data shows most patients were diagnosed at stage 1 while a small percentage were at stage 2 so the diagnosis at an early stage is important in diagnosis and effective treatment which puts the patient at a good prognosis and survival rate [17].

The ATA risk stratification shows most patients were classified as low risk while less than one-third was classified as intermediate risk and a small percentage as high risk. The stratification risk is crucial for post thyroidectomy guiding those with high risk to receive more treatment and follow-up [18].

The RAI was administered in less than half of the individuals. RAI is commonly used in DTC to ablate thyroid residual and treat metastasis. The decision for RAI administration depends on several factors like tumor size, staging, and recurrence risk [19].

This retrospective study analysis of TgAb normalization revealed no significant differences between male and female patients over various time intervals. This result aligns with existing literature, which indicates that gender does not significantly impact TgAb levels post-treatment in DTC patients. Studies have shown that while thyroid cancer is more prevalent in women, gender does not affect the recurrence rates or TgAb dynamics significantly [3,20].

A significant finding of this study is the influence of BMI on TgAb normalization, particularly at the > 48-month interval. Patients with a higher BMI (>30 kg/m^2^) exhibited significantly lower TgAb normalization rates. This observation is consistent with a previous study that links obesity with poorer thyroid cancer outcomes [21]. Higher BMI can create an inflammatory environment, which may interfere with TgAb normalization and overall cancer prognosis [22,23].

The study found no significant impact of age on TgAb normalization, with similar rates observed in patients younger than 55 and those older than 55. This is supported by existing evidence showing that age, while influencing the overall prognosis and aggressiveness of thyroid cancer, does not directly affect the levels of TgAb post-treatment [17,24].

Whether patients underwent lobectomy or total thyroidectomy, this study did not show a significant effect of the type of surgical intervention on TgAb normalization. This finding is in agreement with the current understanding that both surgical methods can effectively manage TgAb levels, provided they are selected based on individual risk and disease characteristics. The choice between lobectomy and total thyroidectomy is often based on the extent of disease and risk factors rather than TgAb normalization potential [17,25,26].

The study observed no significant differences in TgAb normalization between patients with papillary and non-papillary thyroid cancers. These findings are similar to those of previous studies showing that TgAb dynamics are comparable within different types of DTC [24,27].

Furthermore, the risk of recurrence classification according to ATA shows no significant impact on TgAb normalization. This suggests that even patients with a high risk of recurrence can achieve TgAb normalization in a similar way; thus, an effective management strategy should be designed to their risk profile. The finding of this study is comparable with previous studies indicating that risk adapting management approach can reduce the effect of high recurrence risk on TgAb level [28,29].

In this study, the normalization of TgAb between patients who received RAI treatment and those who did not was not significant. This result is comparable with previous results showing that RAI, while effective as an adjuvant therapy, does not uniformly impact TgAb levels. The effectiveness of RAI in reducing TgAb levels may depend on factors such as initial surgical interference and the disease extent [2,20].

The limitations of this study were retrospective design, small sample size, incomplete data, time interval analysis difficulty, uncontrollable confounders, and single-center nature. Furthermore, the management of the patients was not only handled by endocrinologists but also involved general surgeons, nuclear medicine specialists, and internal medicine physicians, emphasizing the importance of teamwork; however, this may lead to overlapping of missing information. Additionally, some patients were initially managed outside our center and were either referred to us by other doctors or came on their own to complete their treatment. Hence, a part of their earlier data may have been missing.

## Conclusions

This study indicates that gender, age, type of surgery, type of cancer, ATA risk of recurrence, and RAI treatment do not significantly affect the normalization of TgAb in DTC patients over time. However, a higher BMI is associated with less consistent TgAb normalization, particularly in the long term. 
